# Acute dapagliflozin administration exerts cardioprotective effects in rats with cardiac ischemia/reperfusion injury

**DOI:** 10.1186/s12933-020-01066-9

**Published:** 2020-06-15

**Authors:** Charshawn Lahnwong, Siripong Palee, Nattayaporn Apaijai, Sirawit Sriwichaiin, Sasiwan Kerdphoo, Thidarat Jaiwongkam, Siriporn C. Chattipakorn, Nipon Chattipakorn

**Affiliations:** 1 Cardiac Electrophysiology Research and Training Center, Faculty of Medicine, Chiang Mai University, Chiang Mai, Thailand; 2 Department of Pharmacology, Faculty of Medicine, Khon Kaen University, Khon Kaen, Thailand; 3 Center of Excellence in Cardiac Electrophysiology Research, Chiang Mai University, Chiang Mai, 50200 Thailand; 4 Cardiac Electrophysiology Unit, Department of Physiology, Faculty of Medicine, Chiang Mai University, Chiang Mai, Thailand

**Keywords:** Heart, Ischemia–reperfusion injury, Sodium-glucose co-transporter 2 (SGLT-2) inhibitors, Dapagliflozin, Mitochondria

## Abstract

**Background:**

A sodium-glucose co-transporter 2 (SGLT-2) inhibitor had favorable impact on the attenuation of hyperglycemia together with the severity of heart failure. However, the effects of acute dapagliflozin administration at the time of cardiac ischemia/reperfusion (I/R) injury are not established.

**Methods:**

The effects of dapagliflozin on cardiac function were investigated by treating cardiac I/R injury at different time points. Cardiac I/R was instigated in forty-eight Wistar rats. These rats were then split into 4 interventional groups: control, dapagliflozin (SGLT2 inhibitor, 1 mg/kg) given pre-ischemia, at the time of ischemia and at the beginning of reperfusion. Left ventricular (LV) function and arrhythmia score were evaluated. The hearts were used to evaluate size of myocardial infarction, cardiomyocyte apoptosis, cardiac mitochondrial dynamics and function.

**Results:**

Dapagliflozin given pre-ischemia conferred the maximum level of cardioprotection quantified through the decrease in arrhythmia, attenuated infarct size, decreased cardiac apoptosis and improved cardiac mitochondrial function, biogenesis and dynamics, leading to LV function improvement during cardiac I/R injury. Dapagliflozin given during ischemia also showed cardioprotection, but at a lower level of efficacy.

**Conclusions:**

Acute dapagliflozin administration during cardiac I/R injury exerted cardioprotective effects by attenuating cardiac infarct size, increasing LV function and reducing arrhythmias. These benefits indicate its potential clinical usefulness.

## Background

Ischemic heart diseases are the major cause of global mortality with more than 15 million cases each year [1]. Acute myocardial infarction (AMI) occurs when any coronary arteries are occluded for a duration of time sufficient to cause cardiac cell death [2, 3]. The current treatment for acute MI is to return blood circulation to the heart, a procedure known as reperfusion. This could be accomplished by surgical procedure and balloon angioplasty via percutaneous coronary intervention [4, 5], or antiplatelet or antithrombotic treatment [6]. However, reperfusion itself can elevate the rate of mortality in patients with AMI by causing myocardial cell death and enhancing the infarct size, a process defined as “ischemia/reperfusion (I/R) injury” [7, 8]. Thus, the treatments which preserve myocardial tissues after cardiac I/R injury may help to protect the heart, both structurally and functionally, in patients with AMI.

Sodium-glucose co-transporter 2 (SGLT-2) inhibitors are a novel class of antidiabetic drugs which prohibit the reabsorption of glucose and sodium from the proximal convoluted tubules, resulting in glycosuria and natriuresis properties [9]. Recently, the EMPA-REG OUTCOME trial reported the cardiovascular benefits of empagliflozin, a SGLT-2 inhibitor, by significantly decreasing the incidence of hospitalization associated with heart failure, cardiovascular-cause death rate and all-cause death rate in diabetic patients with cardiovascular diseases [10]. Although the results from this trial found no significant differences in the rate of AMI between group receiving treatment and placebo [10], there was a possible link highlighted between SGLT-2 inhibitors and cardioprotective effects attenuating AMI severity as evidenced by animal studies [11, 12]. Four-week pretreatment with dapagliflozin could decrease infarct size in rats with obese-insulin resistance which underwent cardiac I/R injury [11]. In a chronic myocardial infarct model in rats, dapagliflozin treatment beginning 1 day after left anterior descending coronary artery (LAD) ligation could decrease myofibroblast infiltration and myocardial fibrosis [12]. Despite its potential benefits on the heart, the cardioprotective effects of acute dapagliflozin administration at the time of cardiac I/R injury has never been investigated. We aimed to study the temporal effects of acute dapagliflozin administration on cardiac function given at 3 different times during cardiac I/R injury in rats. Our hypothesis was that acute dapagliflozin administration given after cardiac ischemia could attenuate cardiac dysfunction in rats with cardiac I/R injury.

## Materials and methods

### Experimental animals

**Fig. 1:**
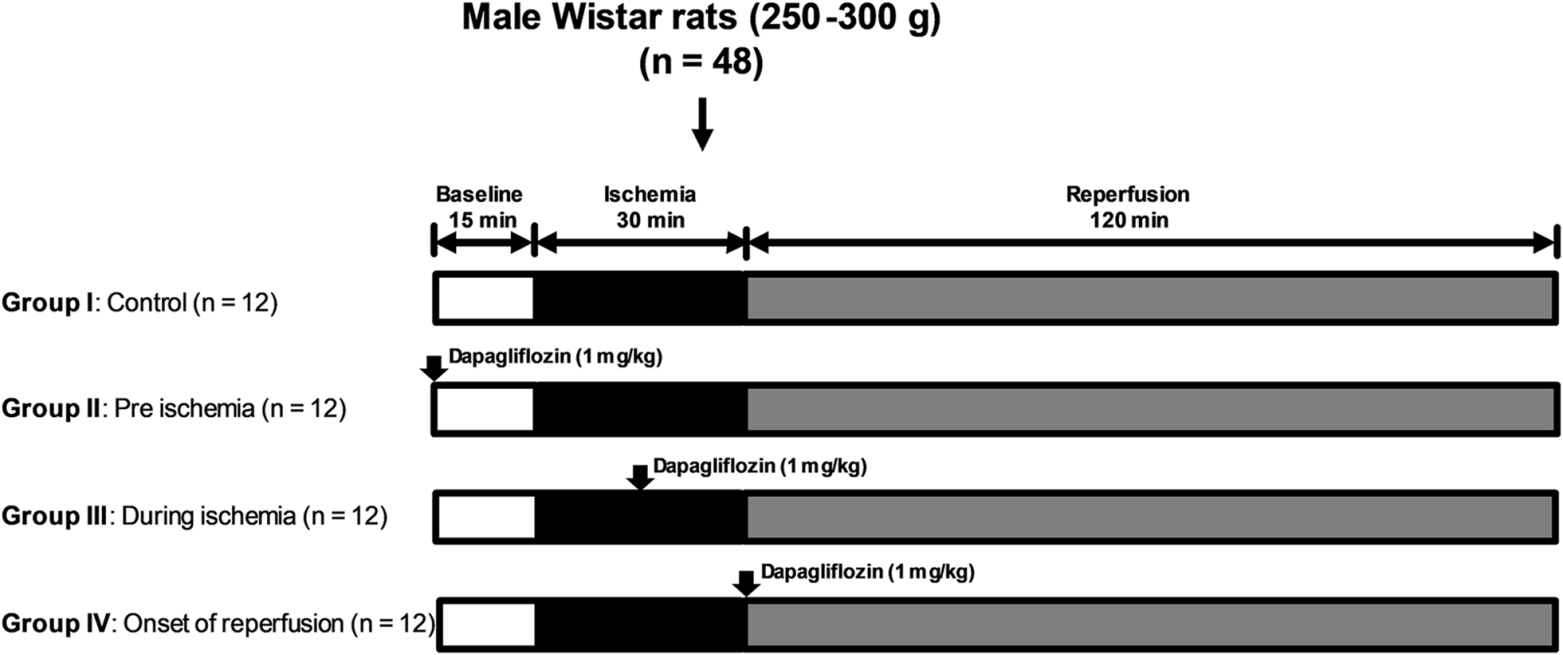
The protocol used to show the dapagliflozin effects on myocardial and mitochondrial function given at pre-ischemic, during ischemic and onset of reperfusion time points during acute I/R injury in rats, *n *= 12 per group. *TTC* triphenyltetrazolium chloride

All experiments in this study were performed in accordance with guidelines for the care and use of laboratory animals by the NIH. The experimental protocol was approved by the Institutional Animal Care and Use Committee, Faculty of Medicine, Chiang Mai University, Chiang Mai, Thailand. After 7 days of acclimatization, male Wistar rats (n = 48, 250–300 g, 8 weeks old) underwent cardiac I/R protocols. These rats were subdivided into four groups to enable 4 different treatments (Fig. 1): (1) Pretreated group: dapagliflozin was administered 15 min before cardiac ischemia; (2) Ischemia group: dapagliflozin was administered 15 min into the cardiac ischemic period; (3) Reperfusion group: dapagliflozin was administered at the onset of reperfusion, and 4) Control group: normal saline solution was administered to the rats as a vehicle. Dapagliflozin (Bristol-Myers Squibb and AstraZeneca Co.) was dissolved in 1 ml of normal saline solution, and 1 mg/kg of dapagliflozin was administered via left femoral vein injection. LV function was determined during the I/R protocol using a pressure–volume (P–V) loop (Transonic, USA) [13]. An arrhythmia score was evaluated from a lead II electrocardiogram (ECG). At the end of the I/R protocol, the heart was excised immediately to enable measurement of myocardial infarct size and the study of myocardial tissues.

### Myocardial I/R

Rats were anesthetized by administration of Zoletil (50 mg/kg, Virbac, Thailand) and Xylazine (0.15 mg/kg, LBS labs, Thailand) intramuscularly. The level of anesthesia was closely monitored by determination of the respiration pattern, eye and pedal reflexes. The rats were ventilated with room air from a rodent ventilator (Cwe, Inc, Ardmore, PA, USA) after the tracheostomy was done. Lead II ECG was recorded during the study using a PowerLab system with Chart 7.0 program (AD Instrument, Australia). A left-side thoracotomy was operated at the fourth intercostal space, the pericardium was cut to expose the heart. The ligation was performed at the left anterior descending coronary artery (LAD) 0.2 cm distal to its origin. The ST segment elevation on lead II ECG and a color change of the myocardial tissue were used to confirm successful ischemia, and the ischemia was continued for 30 min. Then, the ligature was released to induce reperfusion for 2 h [14, 15].

### Determination of arrhythmia parameters and LV function

The incidence of cardiac arrhythmia was determined using the Lambeth Conventions [16]. Arrhythmia scores were evaluated using the criteria described in previous studies [17, 18].

For LV function measurement, the right carotid artery was located and a P–V loop catheter (Transonic, USA) was inserted into the LV to evaluate LV function during the I/R. Heart rate (HR), left ventricular end-systolic and diastolic pressure (LVESP and LVEDP), dP/dt_max_, dP/dt_min_, stroke volume (SV) and left ventricular ejection fraction (LVEF) were determined using a Labscribe program (Labscribe, USA) [13, 14]. For P–V loop data analysis: (1) 50 loops before ischemia were selected to represent the baseline; (2) 50 loops at the end of coronary occlusion were selected to represent the ischemic period, and (3) 50 loops at the end of reperfusion were selected to represent the reperfusion period.

### Infarct size measurement

After 2 h of reperfusion, the rats were sacrificed and the heart was rapidly removed. The LAD was re-occluded and the heart was evaluated the LV area at risk (AAR) by 1 ml Evan’s blue dye perfusion. The heart was kept at − 20 °C overnight and then sectioned horizontally at 1–2 mm thickness. After that, heart slices were immersed in 2,3,5-triphenyltetrazolium chloride (TTC) in phosphate buffer saline solution. The TTC stained area indicated viable tissue which was detected by the red coloration. The infarct size was identified by the white color area that was not stained with any dyes. The myocardial infarct size and the AAR were calculated in accordance with the formula of Reiss et al. using the image tool program [14, 15].

### Cardiac mitochondrial function measurement

The hearts were washed with cold normal saline solution. Cardiac mitochondria were isolated and collected from both remote and ischemic myocardial tissues [19, 20] to determine cardiac mitochondrial function. Variables recorded including cardiac mitochondrial reactive oxygen species (ROS) levels, cardiac mitochondrial membrane potential changes, and cardiac mitochondrial swelling. An enhancement in the 2′,7′-dichlorofluorescein fluorescent intensity demonstrates an increase in mitochondrial ROS production, which correlates with an increase in oxidative stress levels. An attenuation in the red/green fluorescence intensity ratio of JC-1 dye indicates an increase in mitochondrial membrane depolarization. Finally, an attenuation in mitochondrial absorbance at 540 nm implies mitochondrial swelling [19, 20].

### Western blot analysis for mitochondrial biogenesis, dynamics, apoptosis and Connexin 43

The heart was removed and LAD was re-occluded at the same site that had been previously ligated. Then, it was irrigated with normal saline solution through aorta to remove blood inside coronary arteries in remote area, but not ischemic area. Therefore, the ischemia and remote areas could be observed. After that, the tissues from the ischemic and remote areas were separated, chopped into smaller pieces and homogenized in the isolated heart buffer. The homogenate from each area was centrifuged at 800*g* for 5 min to collect the supernatant and then centrifuged it at 8800*g* for 5 min. The pellet was resuspended in the isolated heart buffer and centrifuged at 8800*g* for 5 min. Isolated mitochondria were collected and the protein concentration were determined using a Bradford protein assay (Bio-Rad Laboratories, USA). Proteins sample of 50–80 μg were mixed with the loading buffer and subjected to gel electrophoresis. Then, the protein extracts were transferred to nitrocellulose membranes in a transfer buffer. Membranes were incubated with either 5% skimmed milk or bovine serum albumin in a tris-buffered saline containing 0.1% tween (TBST) for 1 h at room temperature. The membranes were exposed overnight to primary antibodies including PGC1α, CPT1, mitochondrial oxidative phosphorylation complex I-V, DRP1, MFN2 and OPA1 (Cell Signaling Technology, USA) to determine cardiac mitochondrial biogenesis and dynamics. Additionally, the cardiac apoptotic signaling was determined by using the primary antibodies against caspase-3, cleaved caspase-3, Bax and Bcl2 (Cell Signaling Technology, USA). For cardiac gap junction protein assessment, primary antibodies against connexin-43 (Cx43), and p-Cx43 at serine 368 residue (Cell Signaling Technology, USA) were used. Afterwards, the membranes incubated with horseradish peroxidase were conjugated with anti-rabbit IgG (Cell Signaling Technology, USA). Subsequently, western blot bands were detected and used for the analysis of protein expression [14].

### TUNEL-positive cells for evaluation of cardiomyocyte apoptosis

The myocardial apoptosis was determined by terminal deoxynucleotidyl nick-end labeling (TUNEL) assay (Roche, Basel, Switzerland). The cardiac tissue slices were placed in phosphate buffer saline solution for 10 min after dehydration. The slices were incubated with Proteinase k solution (1:50) for 30 min, followed by incubation with CytoninTM for another 120 min. The levels of apoptosis was analyzed as a percentage of the number of TUNEL-positive cells over the total number of DAPI positive cells [21].

### Statistical analysis

The experimental processes and experimental data analyses were carried out following randomization. Data are shown as mean ± SEM. GraphPad Prism 7.0 software was used for data analysis. A one-way-ANOVA followed by an LSD post hoc test were used to test the difference between groups. *p* < 0.05 was considered statistically significant.

## Results

### Dapagliflozin reduced infarct size in cardiac I/R injured rats

**Fig. 2:**
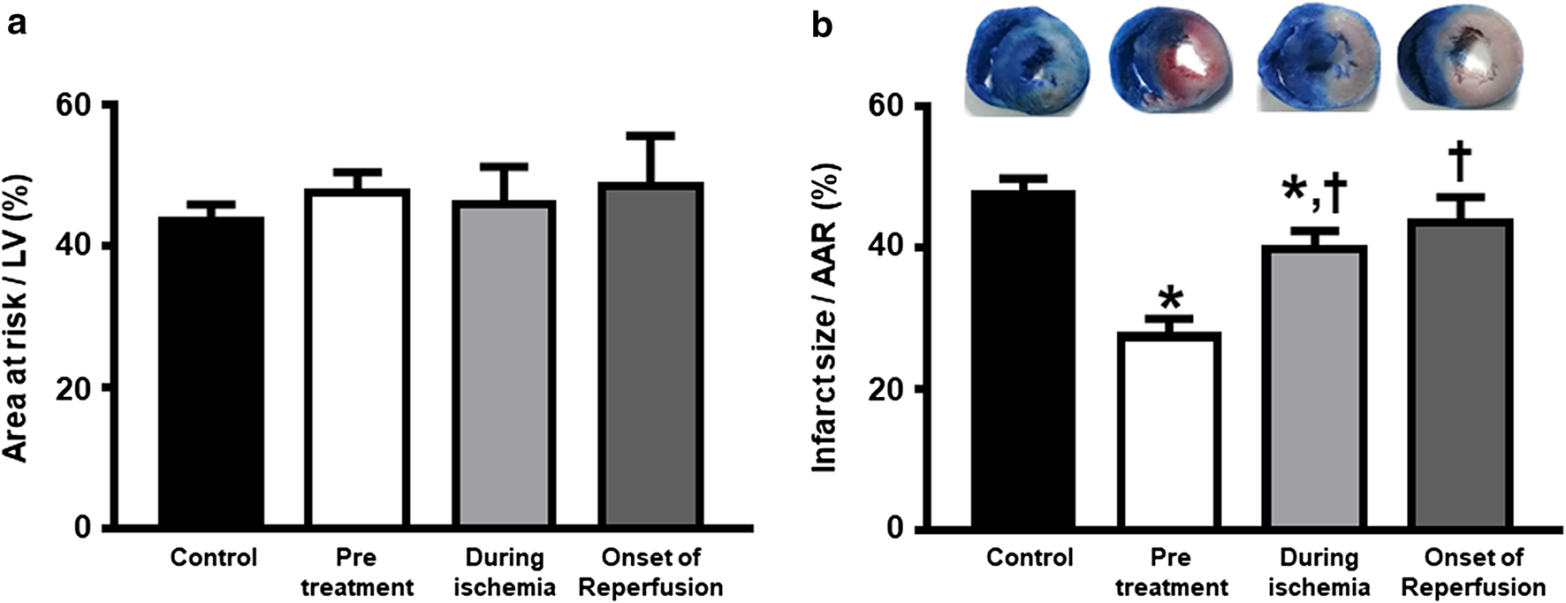
The effects of dapagliflozin on myocardial infarction in rats with cardiac I/R injury. **a** Area at risk, *n *= 6 per group; and **b** Myocardial infarct size, *n* = 6 per group. *p < 0.05 vs control and ^†^*p *< 0.05 vs pretreatment group. *AAR* area at risk

In this study, no mortality was observed during cardiac I/R in any groups. Figure 2 shows the size of myocardial infarction. There is no difference in the area at risk between groups (Fig. 2a). The representative heart sections from the control and the intervention groups are shown in Fig. 2b. Dapagliflozin given at pretreatment and during ischemia significantly reduced the myocardial infarction size, compared to controls (Fig. 2b). However, dapagliflozin given at the onset of reperfusion did not exert this benefit. Differential temporal effects of the drug administration on the infarct size can be clearly observed, the size of infarction in rats pretreated with dapagliflozin showing a reduction of ~ 42% in comparison to the control. This is statistically significantly lower than the sizes in rats treated during ischemia (~ 16% reduction).

### Dapagliflozin reduced cardiac arrhythmia during I/R

**Fig. 3:**
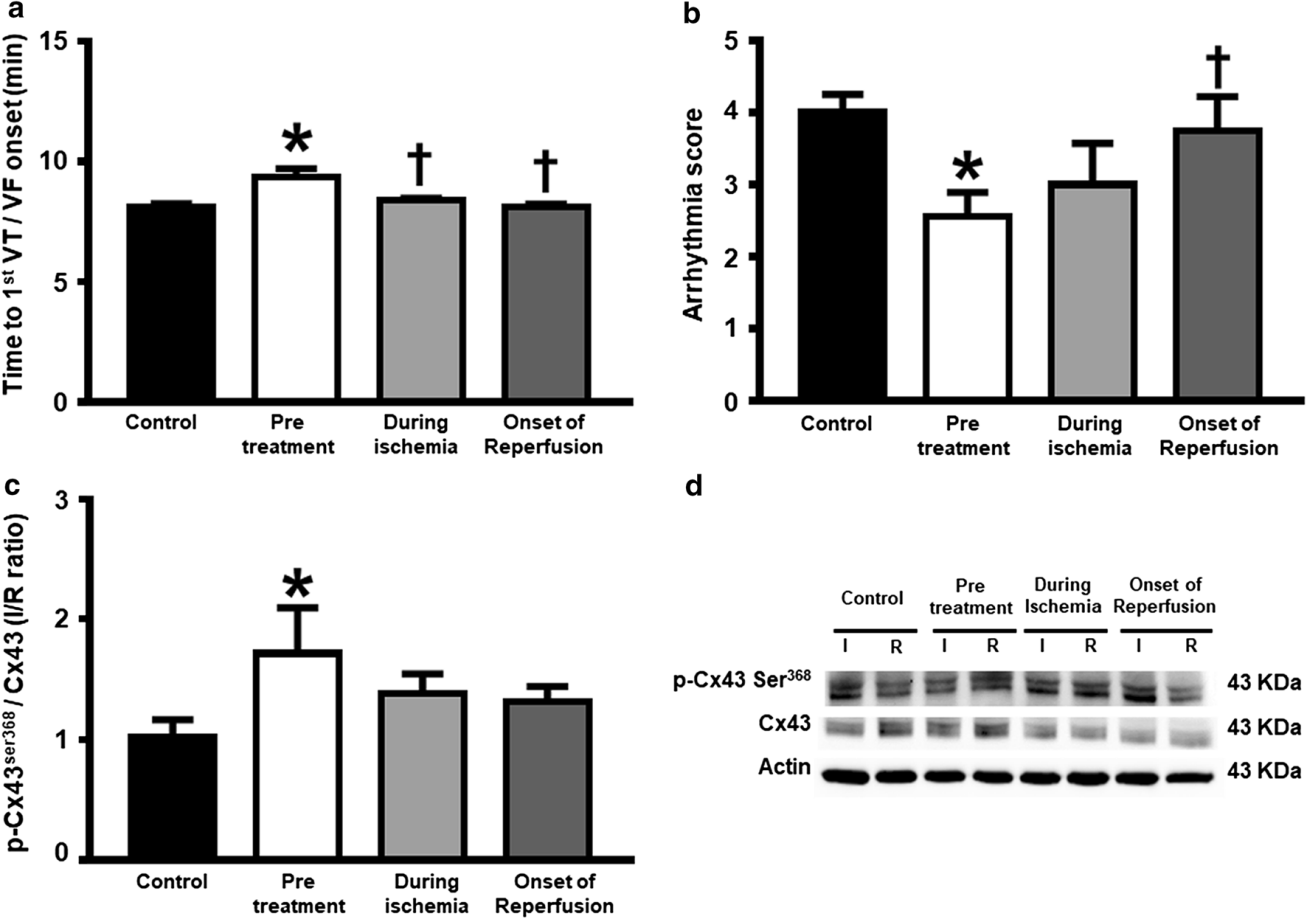
The effects of dapagliflozin on cardiac arrhythmia and gap junction-related proteins in rats with I/R injury. **a** Time to 1st VT/VF, *n* = 6 per group; **b** Arrhythmia score, *n* = 6 per group; **c** p-Cx43 Ser_368_/Cx43 protein expression in the ischemic area normalized with that in the remote area, *n* = 6 per group; and **d** Representative Western blot bands of p-Cx43 Ser368 and Cx43 proteins expression in the ischemic area and the remote area. *p < 0.05 vs control and ^†^*p *< 0.05 vs pretreatment group. *Cx43* connexin, *I* ischemic area, *p-Cx43 Ser638* phosphorylation of Cx43 at serine638, *R* remote area, *VF* ventricular fibrillation, *VT* ventricular tachycardia

Figure 3a, b show that the time to 1st VT/VF onset and arrhythmia score in rats pretreated with dapagliflozin was significantly longer, when compared to rats in the control group. Nevertheless, these parameters in the dapagliflozin treatment during ischemia and at the beginning of reperfusion groups were no different from the control group (Fig. 3a, b). In the pretreated group the ratio of Cx43 phosphorylation (pCx43) to total Cx43 was elevated significantly, compared to the control. This was not the case during ischemia and at the beginning of reperfusion groups (Fig. 3c, d).

### Dapagliflozin improved LV dysfunction during I/R injury

**Fig. 4:**
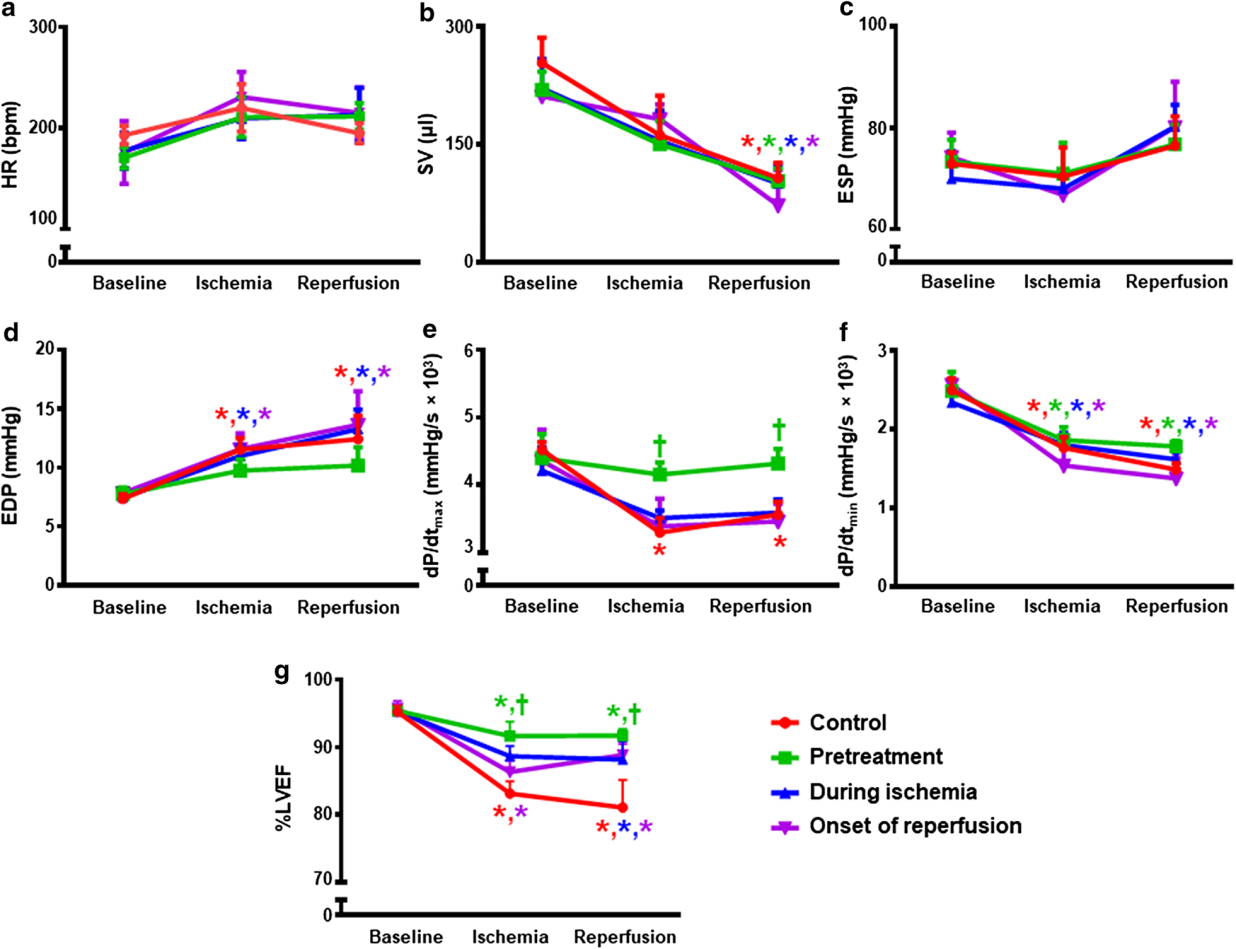
The effects of dapagliflozin on left ventricular function in rats with I/R injury. **a** Heart rate; **b** Stroke volume; **c** Left ventricular end systolic pressure; **d** Left ventricular end diastolic pressure; **e** *dP/dt*_max_; **f** *dP/dt*_min_; and **g** Left ventricular ejection fraction, *n* = 6 per group. *p < 0.05 vs baseline of its group; ^†^p < 0.05 vs control group at that period. *dP/dt* ventricular contractility assessment, *EDP* end diastolic pressure, *ESP* end systolic pressure, *HR* heart rate, *LVEF* left ventricular ejection fraction, *SV* stroke volume

Figure 4a–g shows alteration in LV function at different time points of I/R injury. At the baseline, there was no difference in parameters between groups. After LAD occlusion, an attenuation in stroke volume, *dP/dt*_max_, *dP/dt*_min_ and % left ventricular ejection fraction (LVEF) and an elevation in end diastolic pressure were observed. During ischemic and after reperfusion periods, only the dapagliflozin pretreated group had improved *dP/dt*_max_ and %LVEF, when compared with the control group (Fig. 4e, g). Nevertheless, no improvements were seen in any parameters in the groups where dapagliflozin was given during ischemia and at the beginning of reperfusion.

### Dapagliflozin attenuated cardiac cell apoptosis

**Fig. 5:**
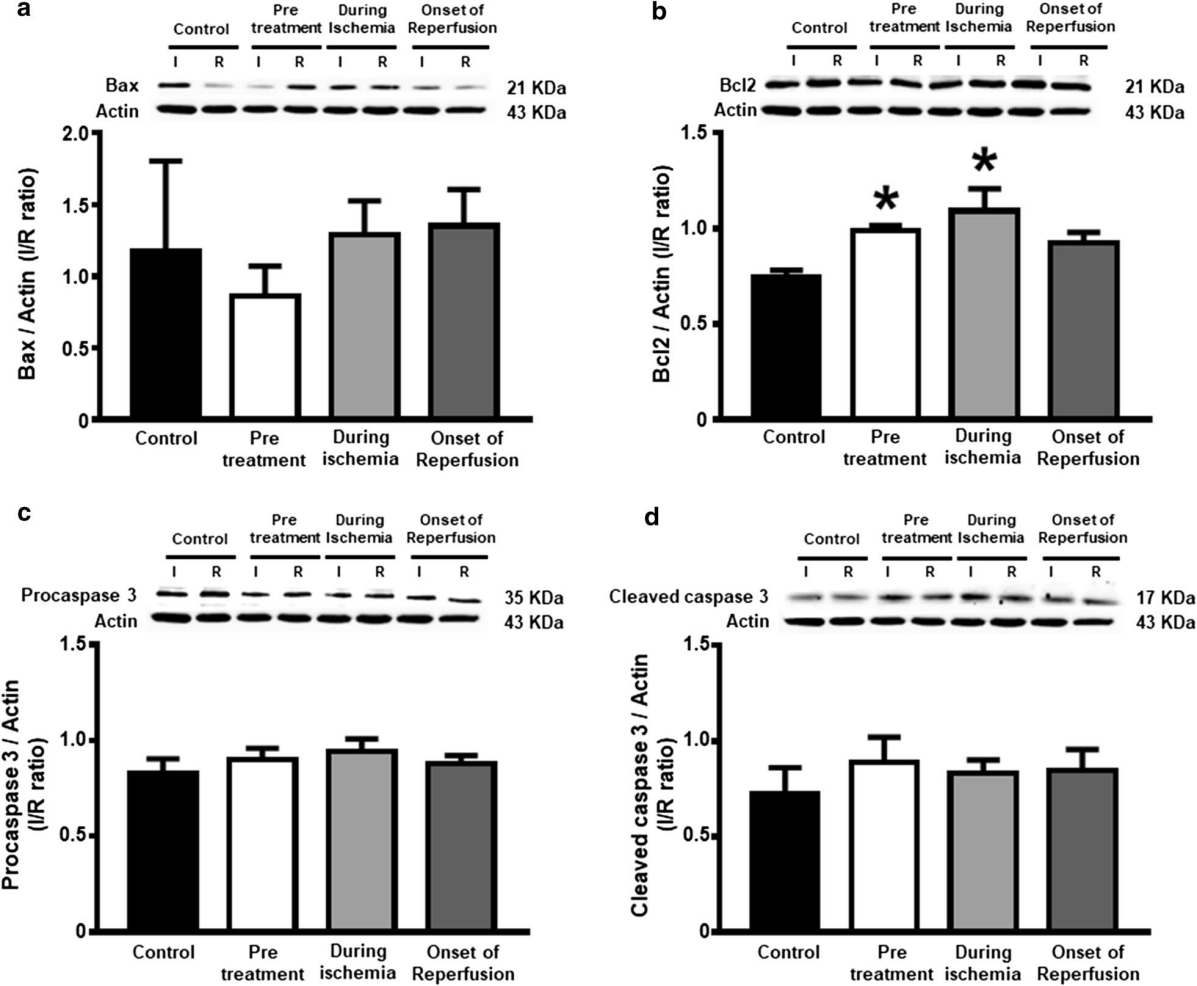
The effects of dapagliflozin on cardiac cell apoptosis in rats with cardiac I/R injury. **a** Bax; **b** Bcl-2; **c** Caspase-3; and **d** Cleaved caspase-3 expression in the ischemic area normalized with that in the remote area, *n* = 6 per group. *p < 0.05 vs control. *I* ischemic area, *R* remote area

**Fig. 6:**
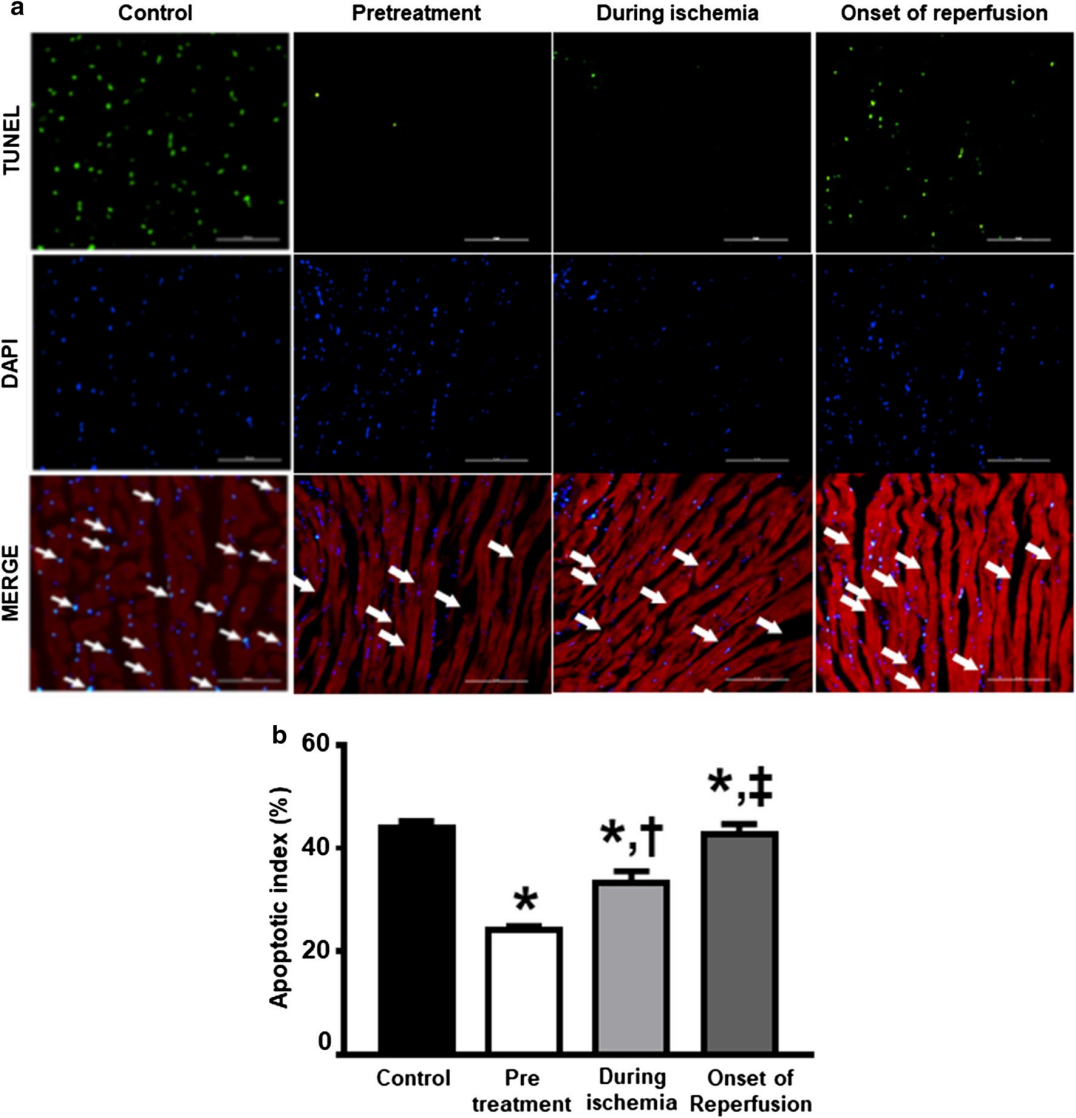
The effect of dapagliflozin on TUNEL-positive cells in the heart. **a** Representative images of TUNEL-positive cells; **b** Apoptotic index, *n* = 4 per group. *p < 0.05 vs control; ^†^p < 0.05 vs pretreatment group; ^‡^p < 0.05 vs during ischemia

The expression of anti-apoptotic protein Bcl-2, pro-apoptotic protein Bax, caspase 3 and cleaved-caspase 3 are shown in Fig. 5a–d. The Bcl-2 levels in the pretreated group and during ischemia group were significantly increased, when compared to the control group (Fig. 5b). However, the Bax, caspase 3 and cleaved-caspase 3 levels were no different across all groups (Fig. 5a, c, d). The pretreated group and during ischemia group had significantly decreased numbers of TUNEL positive cells, when compared to the control (Fig. 6). Moreover, the TUNEL positive cells were notably attenuated in the pretreated group, compared to the during ischemia group (Fig. 6).

### Dapagliflozin improved cardiac mitochondrial function, mitochondrial dynamics and biogenesis in cardiac I/R injured rats

**Fig. 7:**
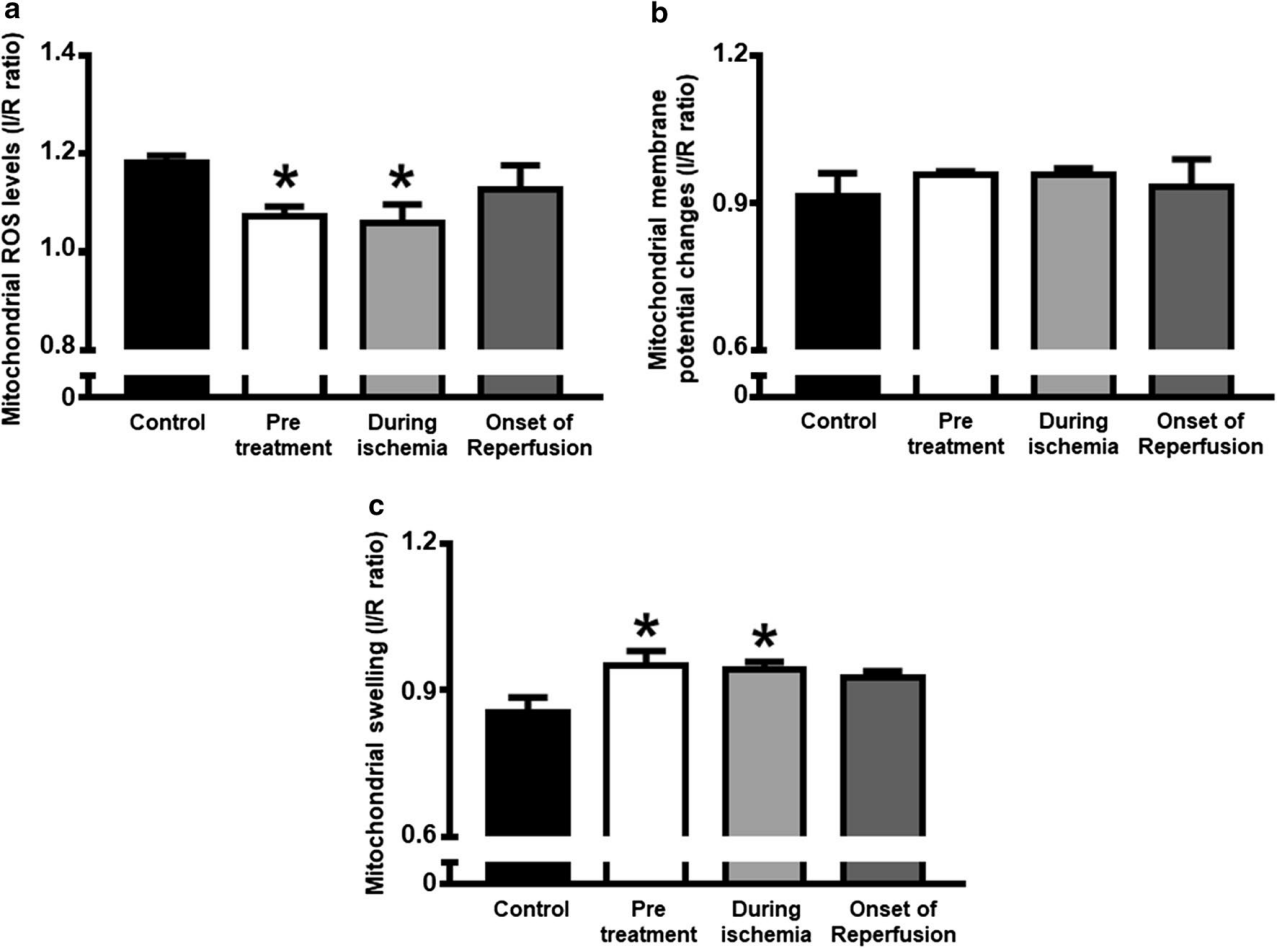
The effects of dapagliflozin on cardiac mitochondrial function in rats with cardiac I/R injury. **a** Mitochondrial ROS production; **b** Mitochondrial membrane potential changes; and **c** Mitochondrial swelling, *n* = 6 per group. *p < 0.05 vs control. *MMP* mitochondrial membrane potential, *ROS* reactive oxygen species

**Fig. 8:**
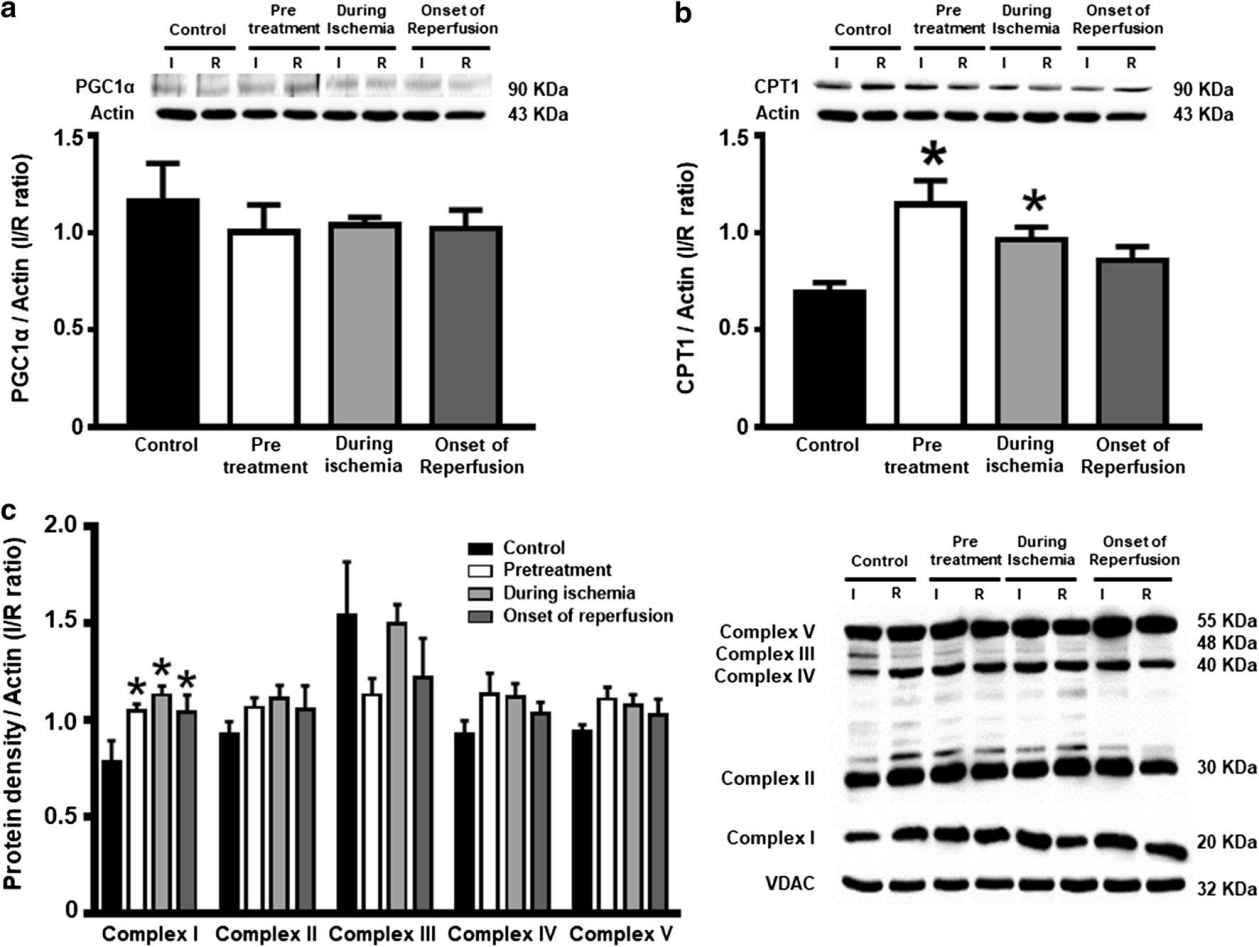
The effects of dapagliflozin on proteins associated with myocardial metabolism and oxidative phosphorylation in rats with I/R injury. **a** Myocardial PGC1-α; **b** Myocardial CPT-1; and **c** Cardiac mitochondrial complex I–V expression in the ischemic area normalized with that in the remote area, *n* = 6 per group. *p < 0.05 vs control. *CPT1* carnitine palmitoyltransferase I, *GAPDH* glyceraldehyde 3-phosphate dehydrogenase, *I* ischemic area, *PGC1-α* peroxisome proliferator-activated receptor gamma coactivator 1-alpha, *R* remote area, *VDAC* voltage-dependent anion channel

**Fig. 9:**
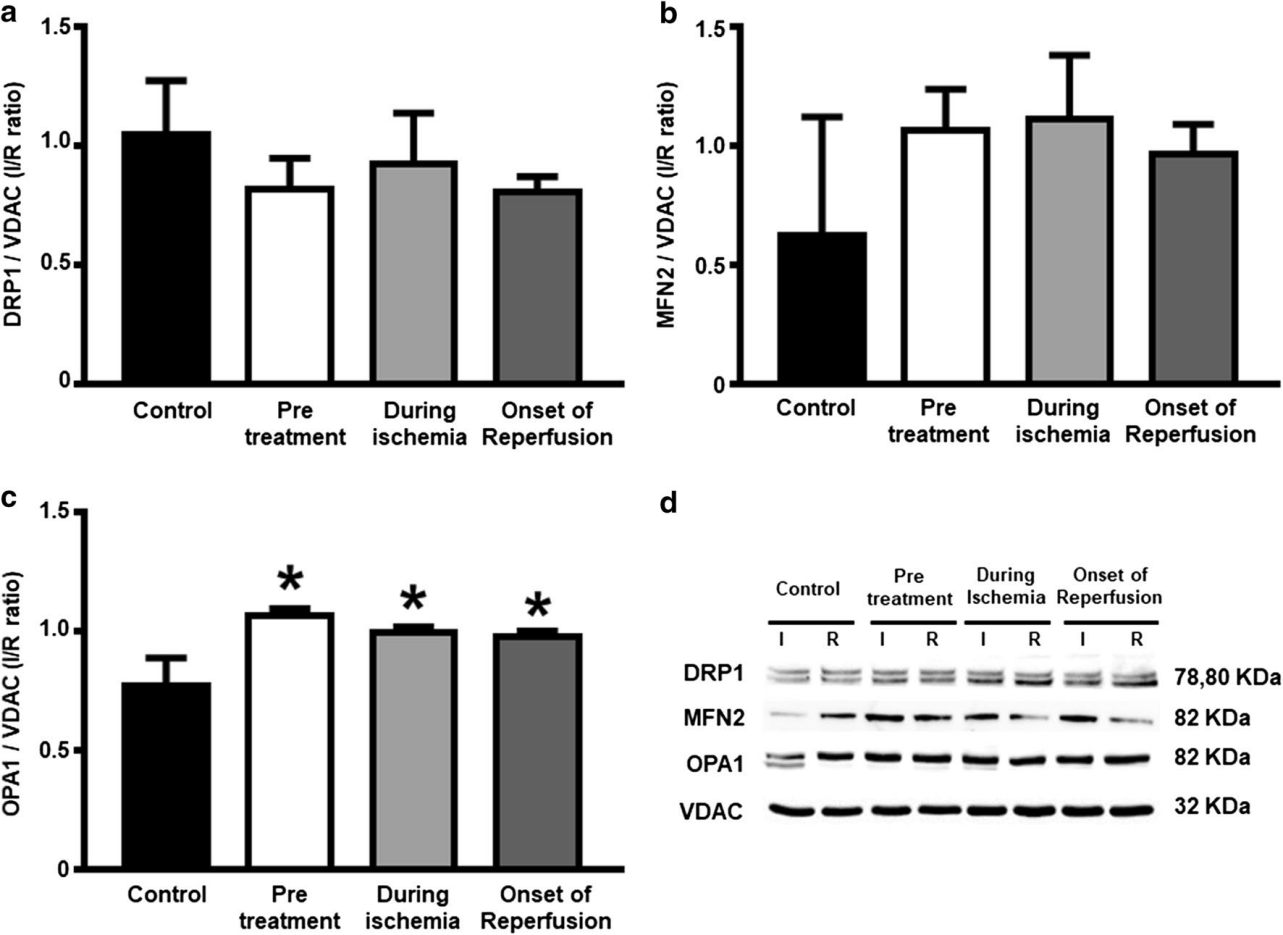
The effects of dapagliflozin on the expression of cardiac mitochondrial fission and fusion protein in rats with I/R injury. **a** Cardiac mitochondrial DRP1; **b** MFN2; **c** OPA1 expression in the ischemic area normalized with that in the remote area, *n* = 6 per group; and **d** Representative western blot bands of **a–c**. *p < 0.05 vs control. *DRP1* dynamin-related protein 1, *I* ischemic area, *MFN2* mitofusin 2, *OPA1* optic atrophy 1, *R* remote area, *VDAC* voltage-dependent anion channel

With regard to mitochondrial function, rats in the pretreated group and during ischemia group had significantly decreased reactive oxygen species (ROS) production (Fig. 7a) and increased absorbance intensity, revealing less mitochondrial swelling (Fig. 7c), compared with the control group. However, the red/green fluorescence intensity ratios, demonstrating mitochondrial depolarization, were no different across all groups (Fig. 7b). These findings were also consistent with the significant elevation in CPT1 observed in the pretreated and during ischemia groups, when compared to the control (Fig. 8b). The level of complex I of the electron transport chain increased significantly in all dapagliflozin treated groups (Fig. 8c), compared to the controls. However, the PGC1-α and complex II-V were no different across all groups (Fig. 8a, d–g). In the case of cardiac mitochondrial dynamics, all treatment groups had similarly increased cardiac mitochondrial OPA1 levels (Fig. 9c). However, the cardiac mitochondrial MFN2 and DRP1 levels were no different across all groups (Fig. 9a, b, respectively).

## Discussion

### The cardioprotective benefits of dapagliflozin from its direct effect

It is known that cardioprotective effects of SGLT-2 inhibitors are due to its systemic effect via glycosuria and natriuresis [22–26]. However, the benefits of dapagliflozin found in this study could be mainly due to its direct effect on the heart since it was given to the rats only 15 min prior to cardiac I/R injury. Therefore, its direct cardiac mechanisms may clarify the explanation for the findings. From present information, the direct effects of SGLT-2 inhibitors occurred through their ability to attenuate ionic dyshomeostasis, mitochondrial dysfunction, oxidative stress, inflammation and apoptosis in the heart [27].

Our results demonstrated that acute dapagliflozin treatment during cardiac I/R injury exerted cardioprotective benefits. The crucial aspects of the data accrued in this study can be summarized as follows: (1) the improvement in LV function shown by increased %LVEF; (2) a decrease in arrhythmia score and an increase in Cx43 phosphorylation; (3) a decrease in size of infarction, and cell apoptosis (TUNEL positive cells) and an increase in Bcl2 causing anti-apoptotic protein expression; (4) an attenuation of mitochondrial dysfunction occurred in the heart as demonstrated by a reduction in swelling and ROS production; (5) an attenuation of cardiac energy metabolism dysfunction evidenced by an increased expression of CPT1 and mitochondrial complex I of the ETC; and (6) an attenuation of cardiac mitochondrial dynamic imbalance as indicated by increased mitochondrial fusion.

### The effect of dapagliflozin on cardiac infarct size and function

Taken together with the EMPA-REG OUTCOME trial, previous clinical studies showed benefits of SGLT-2 inhibitors on cardiac function in patients with type 2 diabetes, ischemic heart and heart failure [28–33]. SGLT-2 inhibitors also reduced myocardial oxidative stress, fibrosis and vascular remodeling which play important roles in the pathogenesis of cardiovascular diseases [34, 35]. Focus on cardiac I/R injury, previous studies have primarily aimed to attenuate size of cardiac infarction [7, 8]. Although previous reports have shown the beneficial effects of dapagliflozin pretreatment during I/R injury by lowering myocardial infarct size [11, 12], its effects given after cardiac ischemia were not known. With regard to the clinical benefits in acute myocardial infarction patients, it is essential that any acute pharmacological intervention must provide cardioprotective effects when it is given after myocardial ischemia. Our study established that acute dapagliflozin treatment during cardiac ischemia could still provide cardioprotective effect by reducing the infarct size (~ 16% reduction), even though it was not as effective as pretreatment (~ 42% reduction). The efficacy of the mechanism used by dapagliflozin for attenuation of the size of infarction will be directly related to its capacity to decrease cardiac mitochondrial dysfunction, enhance mitochondrial fusion and attenuate cardiomyocyte apoptosis. Taken together, all of the above benefits of dapagliflozin treatment could explain the improvement in LV dysfunction seen in this study.

In addition to the infarct size and LV function improvement, our results also showed that rats in the dapagliflozin pretreatment group had a delayed time to first VT/VF onset and lower arrhythmia score, when compared to controls. These findings indicated that dapagliflozin exerted an anti-arrhythmic effect during cardiac I/R injury. In addition, its anti-arrhythmic actions could be explained by its effect on Cx43, which is a cardiac gap junction protein promoting cardiac cell communication through electrical current flow [36]. Cx43 at serine 368 phosphorylation has been shown to increase the translocation of Cx43 to the cell membrane, helping with gap junction formation [36]. In this study, dapagliflozin improved gap junction function as evidenced by an increased pCx43 Ser_368_/Cx43 ratio. All of these effects of dapagliflozin could be reasonably assumed to be related to the reduction in arrhythmia found in this study.

### The effect of dapagliflozin on cardiac mitochondria

It is known that cardiac I/R injury is associated with mitochondrial damage and cardiac cell apoptosis [11, 14]. The mitochondrial damage can occur after 20 min of occlusion and the severity will gradually increase during the reperfusion period [37, 38]. Chronic dapagliflozin treatment for 1 month before cardiac I/R injury in rats with metabolic syndrome lowered the enhancement of mitochondrial ROS production, depolarization and swelling [11]. Our results indicated that the cardiac mitochondrial dysfunction indicated by the increase in mitochondrial ROS production and swelling from cardiac I/R injury were significantly reduced in the acute dapagliflozin pretreatment and during ischemia groups. In the case of mitochondrial biogenesis, PGC1-α and CPT1, which are cardiac mitochondrial metabolism-related proteins, are vital proteins playing roles in cardiac fatty acid oxidation [39, 40]. Our results showed that dapagliflozin treatment increased CPT1 protein expression. Dapagliflozin treatment also elevated complex I of the ETC expression, indicating its action in attenuating the depletion of cardiac energy metabolism when I/R injury occurred.

The maintenance of the number of mitochondria is preserved by the equilibrium between fission and fusion [41]. These cycles keep mitochondria functional by abolishing impaired mitochondria and promoting apoptosis when exposed to stress [42]. The proteins which play roles in helping fusion of the outer and inner membranes are Mitofusin 2 (MFN2) and optic atrophy 1 (OPA1) respectively, whereas the protein which plays roles in membrane constriction during fission is dynamin-related protein 1 (DRP1) [43]. It is known that cardiomyocytes can be preserved during I/R injury and cardiac arrest by using mitochondrial fission inhibitors [44, 45]. A previous study demonstrated that 1-month of dapagliflozin treatment lowered the elevation of cardiac mitochondrial fission and the attenuation of mitochondrial fusion as evidenced by decreased DRP1 and increased MFN2 and OPA1 protein expression in metabolic syndrome rats subjected to cardiac I/R injury [11]. In contrast to chronic treatment, acute administration of dapagliflozin caused no change in the expression of DRP1, a mitochondrial fission protein. However, it increased the expression of OPA1, a mitochondrial fusion protein. Our study showed the benefits of dapagliflozin on mitochondrial dynamics, related expression of proteins, biogenesis and function. These could be the key factors responsible for infarct size attenuation with dapagliflozin treatment.

## Conclusion

Acute dapagliflozin administration during cardiac I/R injury exerted cardioprotective effects by attenuating cardiac infarct size, increasing LV function and reducing arrhythmias. The mechanisms responsible for these benefits are due to an attenuation of apoptotic cardiomyocytes, cardiac mitochondrial dysfunction, cardiac energy metabolism dysfunction and cardiac mitochondrial dynamic imbalance. Therefore, these findings could provide significant insights for future clinical investigations of acute dapagliflozin treatment in acute MI patients.

## Data Availability

All data are provided and available in this manuscript.

## Abbreviations

AAR: Area at risk
AMI: Acute myocardial infarction
Cx43: Connexin-43
CPT1: Carnitine palmitoyltransferase I
DRP1: Dynamin-related protein 1
ECG: Electrocardiogram
EDP: End diastolic pressure
ESP: End systolic pressure
GAPDH: Glyceraldehyde 3-phosphate dehydrogenase
HR: Heart rate
I: Ischemic
I/R: Ischemia/reperfusion
LAD: Left anterior descending coronary artery
LV: Left ventricular
LVEDP: Left ventricular end-diastolic pressure
LVEF: Left ventricular ejection fraction
LVESP: Left ventricular end-systolic pressure
MFN2: Mitofusin 2
MMP: Mitochondrial membrane potential
OPA1: Optic atrophy 1
pCx43: Connexin-43 phosphorylation
PGC1-α: Peroxisome proliferator-activated receptor gamma coactivator 1-alpha
P–V: Pressure–volume
R: Remote
ROS: Reactive oxygen species
SGLT-2: Sodium-glucose co-transporter 2
SV: Stroke volume
TBST: Tris-buffered saline containing 0.1% tween
TUNEL: Terminal deoxynucleotidyl nick-end labeling
TTC: Triphenyltetrazolium chloride
VDAC: Voltage-dependent anion channel
VF: Ventricular fibrillation
VT: Ventricular tachycardia
